# Association of rs3798220 Polymorphism with Cardiovascular Incidents in Individuals with Elevated Lp(a)

**DOI:** 10.3390/diagnostics15040404

**Published:** 2025-02-07

**Authors:** Dunja Leskovar Lemešić, Livija Šimičević, Lana Ganoci, Andrea Gelemanović, Nediljko Šućur, Ivan Pećin

**Affiliations:** 1 Division for Metabolic Diseases, Department of Internal Medicine, University Hospital Centre Zagreb, 10000 Zagreb, Croatia; nsucur@gmail.com (N.Š.); ivanpecin@yahoo.com (I.P.); 2 Division for Pharmacogenomics and Therapy Individualization, Department of Laboratory Diagnostics, University Hospital Centre Zagreb, 10000 Zagreb, Croatia; lana.ganoci@kbc-zagreb.hr; 3 Department of Medical Chemistry, Biochemistry and Clinical Chemistry, School of Medicine, University of Zagreb, 10000 Zagreb, Croatia; 4 Department of Basic and Clinical Pharmacology, School of Medicine, University of Zagreb, 10000 Zagreb, Croatia; 5 Mediterranean Institute for Life Sciences (MedILS), University of Split, 21000 Split, Croatia; andreagelemanovic@gmail.com; 6 Department of Internal Medicine, School of Medicine, University of Zagreb, 10000 Zagreb, Croatia

**Keywords:** cardiovascular diseases, cardiovascular risk factors, case–control studies, lipoprotein(a), single nucleotide polymorphism

## Abstract

**Background/Objectives**: Lipoprotein (a) [Lp(a)] plays a significant role in atherosclerosis and cardiovascular disease (CVD). Genetic regulation of Lp(a) involves variations in the apo(a) *LPA* gene, as specific polymorphisms like rs10455872 and rs3798220, both linked to higher Lp(a) levels and CVD. CVD remains the leading global cause of death, with high Lp(a) levels increasingly recognized as a significant factor in younger patients with no other CVD risk factors. We aimed to evaluate the association of *LPA* genetic variations with Lp(a) levels and its effect on cardiovascular risk as there are existing inconsistent findings. **Methods**: This case–control study included 251 subjects with a median age of 52 years (interquartile range, IQR = 17) and elevated Lp(a) levels. Cases were subjects who experienced early cardiovascular incidents (women < 65, men < 55 years old), and the control group included subjects without such history. Genotyping of *LPA* gene polymorphisms (rs10455872 and rs3798220) was performed, and demographic data with Lp(a) levels were collected. To evaluate the association between the *LPA* genotypes and the risk of cardiovascular incidents (CVI), several logistic regression models were performed. The cut-off points for Lp(a) levels were determined using diagnostic test accuracy measures. **Results**: The rs3798220-*C* allele was associated with higher Lp(a) levels (288 ± 166 nmol/L in cases vs. 189 ± 102 nmol/L in controls, *p* < 0.001) and myocardial infarction (53% in cases vs. 36% in controls, *p* = 0.036). Among cases, 28.9% carried the rs3798220-*C* allele, compared to 18.7% in controls. The rs10455872-G allele was slightly more prevalent in controls (34.15% vs. 29.69%) but without further significant associations. In this study, the cut-off Lp(a) value of 151 nmol/L, for patients with a positive family history of early CVD, is associated with a higher chance of developing CVI. **Conclusions**: This study demonstrates an association between the *LPA* rs3798220-*C* allele and higher Lp(a) levels, as well as an increased risk of early onset myocardial infarction. However, the obtained association should further be evaluated at a much larger scale.

## 1. Introduction

Cardiovascular diseases (CVD) are the leading cause of death in Europe with more than 3 million deaths per years, although mortality rates have declined by more than 50% in the last decades [1]. According to data for 2018, 43.7% of the total number of deaths in Croatia was a result of CVD [2]. Among others traditional risk factors (obesity, metabolic syndrome, smoking, arterial hypertension, etc.), dyslipidaemia plays a major role in the development of atherosclerotic disease.

Lipoprotein (a) [Lp(a)] is a low-density lipoprotein (LDL)-like lipid particle containing apolipoprotein (a) [apo(a)], which has prothrombotic and proinflammatory properties. It promotes atherosclerosis by binding oxidized and interacting with various receptors, though it lacks a specific one [3]. Observational and genetic evidence convincingly demonstrates that a high Lp(a) level is causal for atherosclerotic cardiovascular disease (ASCVD) and all-cause mortality, affecting around 1.4 billion people worldwide [4]. Elevated Lp(a) levels (>75 nmol/L or >30 mg/dL) increase cardiovascular risk up to 2–3-fold, contributing to the occurrence of cardiovascular incident (CVI)-like myocardial infarction (MI), ischaemic stroke, peripheral arterial disease (PAD), or aortic valve stenosis [5].

Although several conditions (e.g., chronic kidney or liver disease and autoimmune diseases) influence Lp(a) levels, they are largely genetically determined (90% heritability) and not significantly modifiable by lifestyle changes. Conventional lipid-lowering therapies (statins, ezetimibe, proprotein convertase subtilisin/kexin type 9 [PCSK 9] inhibitors, etc.) have little effect on Lp(a) levels but are often prescribed as a risk mitigation strategy [6,7]. Newer RNA-based therapies show promising results on the reduction in Lp(a) levels but still remain in clinical trials [8,9,10]. Currently, Lp(a) apheresis is the only available treatment, reserved for high-risk patients only due to its invasiveness [11].

The Lp(a) gene [*LPA*] on chromosome 6(q) encodes apo(a) and contains a signal peptide, two types of kringle domains (IV and V) and a protease-like domain [12]. The number of kringle IV type-2 (KIV-2) repeats inversely correlates with the Lp(a) concentration and explains most of the variability (30–70%) in Lp(a) concentration, but there are other gene variations that affect the levels of Lp(a) [13]. Frequent and rare functional single nucleotide polymorphisms (SNPs) modify Lp(a) isoform size and concentrations. The rs10455872 is an intron variant (NC_000006.12:g.160589086A>G) with a minor allele frequency of 7% while the rs3798220 (NC_000006.12:g.160540105T>C) is in the region that encodes the protease-like domain of apo(a) with a frequency of 1.8% in European (non-Finnish) populations [14]. Both SNPs are associated with coronary disease and higher levels of Lp(a), though prevalence and effect size can vary across populations [15,16].

Despite strong genetic influence, the relationship between Lp(a) levels and CVD risk remains complex. Some individuals with mild Lp(a) elevations (2-fold) develop severe atherosclerosis, while others with extremely high levels (>430 nmol/L) remain disease-free. Understanding genetic predisposition could improve cardiovascular risk stratification [17]. Therefore, timely detection of high-risk polymorphisms in individuals with high Lp(a) levels, with treatment and follow-up, might prevent cardiovascular events.

In this study, we aimed to evaluate the association of *LPA* rs10455872 and rs3798220 polymorphisms with Lp(a) levels and its effect on cardiovascular risk as existing published data are not consistent.

## 2. Materials and Methods

This study protocol was approved by the institutional Ethics Committees: University of Zagreb, School of Medicine (Reg. No. 380-59-10106-22-111/86; Class 641-01/22-02/01) and University Hospital Centre Zagreb (Protocol Code: 8.1.-21/158-2, No: 02/21-JG). This study was performed according to the Declaration of Helsinki. The signed informed consents were obtained from all participants.

### 2.1. Subjects

A total of 251 subjects aged 18–65 years with elevated levels of Lp(a) were included in our case–control study over a period of 3 years (2021–2024) at the University Hospital Centre Zagreb, Croatia. The elevated levels of Lp(a) were defined as the following: Lp(a) > 75 nmol/L [18]. The main exclusion criteria were the following: subjects aged over 65 or below 18 years, chronic kidney disease (estimated glomerular filtration rate [eGFR] < 60 mL/min/m^2^), active smoking status (smoking cigarettes continuously, at least one cigarette per day, for at least one year), acute infection (elevated inflammatory parameters: C-reactive protein [CRP] and erythrocyte sedimentation rate [ESR]), history of autoimmune disease and other chronic or malignant disease. Diagnoses of familial hypercholesterolemia or other hereditary dyslipidemia were not a part of the exclusion criteria. The case group consisted of subjects who developed CVI at an earlier age (men < 55 and women < 65 years), and the control group consisted of subjects without a history of CVI. CVI was classified as the following: myocardial infarction, ischemic cerebral stroke and PAD (defined as ankle-brachial index ≤ 0.90 [19]). General demographic data, cardiovascular history and biochemical analysis of Lp(a) were collected from each subject’s medical data as well as a blood sample for *LPA* genotyping. The Lp(a) was measured on an Alinity c clinical chemistry system (Abbott Laboratories, Chicago, IL, USA) with a Randox Lp(a) assay standardised to the WHO/IFCC (IFCC SRM 2B) (Randox Laboratories, Crumlin, UK).

### 2.2. Genotyping

DNA was extracted from 3 mL of whole blood taken in a K_3_EDTA tube (Vacuette, Greiner BioOne International AG, Kremsmünster, Austria) using a FlexiGene DNA Kit (Qiagen, Hilden, Germany). Genotyping of the *LPA* gene polymorphisms (rs10455872 and rs3798220) was performed using the real-time PCR method SNP Genotyping by specific TaqMan^®^ assays ID (C__30016089_10 and C__25930271_10) (Applied Biosystems, Thermo Fisher Scientific, Waltham, MA, USA), on a ABI 7500 Real-Time PCR System (Applied Biosystems, Thermo Fisher Scientific, Waltham, MA, USA).

### 2.3. Statistical Analysis

The number of included subjects was determined on the basis of an a priori analysis in which, with the power analysis of the study is 0.8, the level of statistical significance α = 0.05 (Figure S1). Categorical variables were presented as frequencies and percentages, while numerical variables were first tested for normality using the Shapiro–Wilk test and presented with medians and an interquartile range (IQR) when deviated from normality. To test the difference between the two groups (cases vs. controls or depending on genotype class), a chi-square test was used for categorical variables, whereas the Mann–Whitney U test was used for numerical variables. To evaluate the association between the *LPA* genotypes and the risk of CVI, univariate, multivariate and stepwise logistic regression models were performed. A multivariate logistic regression was adjusted for Lp(a) levels, whereas all measured variables were entered as independent variables in stepwise logistic regression models. The results of logistic regression models were presented with odds ratios and 95% confidence intervals (CI). The statistical significance was set at *p* < 0.05. To provide potential future guidelines for testing the presence of *LPA* genotypes as a risk factor for CVI, cut-off points for Lp(a) levels were calculated based on diagnostic test accuracy measures (sensitivity and specificity). Complete analyses were performed in the free software environment for statistical computing R version 4.3.2 (R Foundation for Statistical Computing, Vienna, Austria) [20], with the following additional packages: ggplot2 v3.5.1 [21] and ggpubr v0.6.0 [22] for visualizations, while cutpoint v1.2.0 [23] for calculating cut-off points for Lp(a) levels.

## 3. Results

### 3.1. Demographic and Medical Data

Of the 251 subjects, with a median age of 52 years (IQR = 17), 42% were women. Comparison between 128 individuals with (cases) and 123 without (controls) a history of an early CVI is shown in Table 1. In short, individuals in the cases group were predominantly male (68%), of older age (57 ± 15 years) and with higher prevalence of type 2 diabetes mellitus (20% vs. 8%) than the control group. The median level of Lp(a) in the cases group was 228.5 nmol/L (IQR = 142) and 189 nmol/L (IQR = 120.5) in the control group. The distribution of cardiovascular events in cases group was as follows: myocardial infarction (79%), ischemic cerebral stroke (21%) and PAD (18%). Only seven individuals in the cases group had aortic stenosis recorded, while none in the control group. Both groups have almost equal prevalence of a positive family history of early CVI (around 80%).

### 3.2. LPA rs3798220 and rs10455872 Genotyping Data

Genotyping of *LPA* gene rs3798220 in a group of cases revealed that 30% were *C*-variant allele carriers, of which one subject is homozygous (*CC*)—a 32-year-old male who had a myocardial infarction and whose Lp(a) levels > 500 nmol/L. In the control group, the prevalence of the rs3798220-*C* allele was 19%. On the other hand, the rs10455872-*G* allele had a slightly higher prevalence in the control group (34%) compared to a group of cases (30%), while none of the subjects were homozygous for the rs10455872-*G* allele. When evaluating the prevalence of both rs3798220-*C* and rs10455872-*G*, only five and three individuals were heterozygous for both SNPs in the cases and control group, respectively. Despite these slight differences in minor allele prevalence, neither rs3798220-*C* nor rs10455872-*G* reached a statistically significant difference between the two groups (Table 1). It is important to emphasize that, in the group of cases who had a myocardial infarction, the *LPA* rs3798220-*C* allele was more prevalent than in those subjects who did not have a myocardial infarction. None of such associations were found for the *LPA* rs10455872 genotype.

### 3.3. Association Between Lp(a) Levels and LPA Genotypes

**Figure 1:**
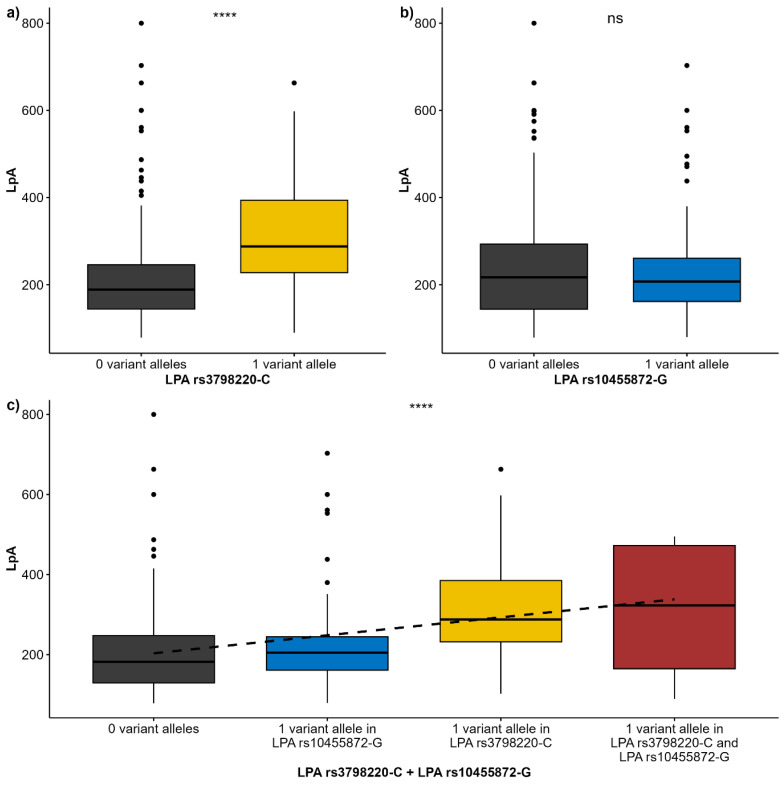
Association between Lp(a) levels and LPA genotypes. (**a**) LPA rs3798220-C; (**b**) LPA rs10455872-G; (**c**) joint prevalence of LPA rs3798220-C and LPA rs10455872-G. Abbreviations: **** *p* < 0.0001, ns—not significant (*p* > 0.05).

When evaluating the association between Lp(a) levels and *LPA* genotypes, it is evident that Lp(a) level increases with the number of *C* alleles in *LPA* rs3798220 (*p* < 0.001), while no difference is found for the *G* allele rs10455872 (Figure 1a,b). As expected, a positive trend is observed when evaluating joint prevalence of both *LPA* genotypes (Figure 1c).

**Figure 2:**
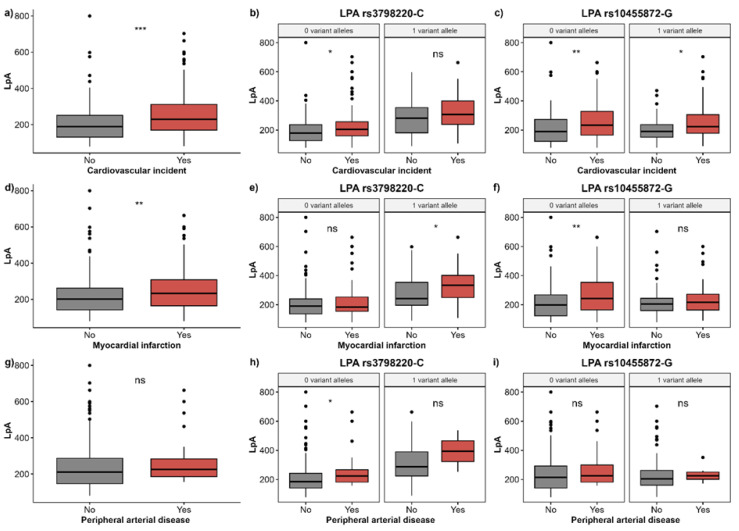
(**a**) Association between Lp(a) levels and prevalence of all cardiovascular incidents (first row), myocardial infarction (second row) or peripheral arterial disease (third row), among all study participants (**a**,**d**,**g**) or upon stratification by number of variant alleles: *LPA* rs3798220-*C* allele (**b**,**e**,**h**) or *LPA* rs10455872-*G* allele (**c**,**f**,**i**). Abbreviations: *** *p* < 0.001, ** *p* < 0.01, * *p* < 0.05, ns—not significant (*p* > 0.05).

A more detailed evaluation of Lp(a) levels showed that Lp(a) levels are significantly increased in the group of cases with CVI and in individuals with a myocardial infarction, whereas this trend is observed in individuals with peripheral arterial diseases, however without reaching statistical significance (Figure 2a,d,g). After stratifying individuals based on their *LPA* genotypes, it is evident that the trend of increased Lp(a) levels remains (Figure 2b,c,e,f,h,i). For the *LPA* rs3798220-*C* allele, the strongest association is observed with prevalence of a myocardial infarction (Figure 2e). Interestingly, for the *LPA* rs10455872-*G* allele, the strongest observation is observed with the overall prevalence of CVI (Figure 2c).

To evaluate this observed association between the *LPA* genotypes and the risk of CVI more in-depth, several logistic regression models were performed (Table 2). First, for each observed outcome (total CVI, myocardial infarction and peripheral arterial disease), univariate logistic regression was performed to evaluate the association of each *LPA* variant with the outcome, independently and adjusted for age since individuals in the CVI group were statistically older than the controls [24]. Next, a multivariate logistic regression was performed to evaluate the effect of both *LPA* SNPs and Lp(a) levels with the outcome, independently and adjusted for age. Finally, stepwise logistic regression models were performed where all other collected variables were used as covariates. When evaluating the CVI as an outcome, *LPA* rs3798220-*C* was associated with an increased risk; however, this result was not replicated after an adjustment for Lp(a) levels. In the stepwise regression, the strongest positive predictors for CVI were male gender, older age and increased levels of Lp(a). When stratifying for myocardial infarction, *LPA* rs3798220-*C* was again shown to be a significant positive predictor, which was again abolished after Lp(a) adjustments. However, in the stepwise regression model, *LPA* rs3798220-*C* remained as a meaningful contributor to an increased risk for myocardial infarction. Even though it did not reach statistical significance in the final stepwise model, it remained in the model, which highlights that this predictor is important in explaining the overall relationship with the increased risk for myocardial infarction.

Finally, when looking at PAD, *LPA* rs3798220-*C* was identified with decreased risk, even after Lp(a) adjustment and in the final stepwise model which included older age, increased levels of Lp(a) and presence of type 2 diabetes mellitus (T2DM). The *LPA* rs10455872-*G* allele was not identified as a significant risk factor in any of the models. After adjusting the univariate and multivariate regression models for age, all the obtained results remained the same, as they were in the models with age as a covariate.

In addition, we performed a multivariate linear regression adjusted for age and a stepwise linear regression analysis to evaluate the contribution of LPA genotypes towards Lp(a) concentration levels, and as expected the *LPA* rs10455872-*G* did not show any statistical association. However, the addition of the *LPA* rs3798220 C allele was shown to increase Lp(a) concentration by 102.44 ± 17.56 nmol/L (*p* < 0.0001).

Finally, to provide potential future guidelines for testing the presence of *LPA* rs3798220-*C* as a risk factor for CVI, cut-off points for the Lp(a) levels were calculated based on diagnostic test accuracy measures (sensitivity and specificity). Cut-off points for the Lp(a) levels were calculated for each outcome individually and when stratifying based on family history of early CVI or with *LPA* rs3798220 genotype. Results are presented in Table 3.

## 4. Discussion

This case–control study on a very homogeneous Croatian population of Caucasian origin showed significant association of the *LPA* rs3798220-*C* allele (*TC* and *CC* genotype) with elevated levels of Lp(a) and myocardial infarctions at an early age of onset (men < 55 and women < 65 years). Comparing both groups of subjects according to their *LPA* rs3798220 genotype, significant association was found only between higher levels of Lp(a) (288 ± 166 nmol/L in cases vs. 189 ± 102 nmol/L in controls and *p* < 0.001) and myocardial infarction (53% in cases vs. 36% in controls and *p* = 0.036). However, for the *LPA* variant rs10455872, our study found no association between higher levels of Lp(a) or CVI. Our study presents a cohort of younger patients, for whom Lp(a) concentration is the most significant risk factor. The results identified male gender as a strong predictor of CVI pointing to the general observation that males are more likely to develop CVD [25,26]. Since our study could not be fully matched in the terms of gender, we kept this variable as independent in the regression model.

It is well known that several *LPA* variants greatly affect LP(a) concentrations and cardiovascular risk. In the European population, rs10455872 and rs3798220 polymorphisms are more common and correlate with higher Lp(a)-related cardiovascular risk. Despite this, the results of the published studies vary. The study of Robert Clark et al. on over 6.000 subjects from the Precocious Coronary Artery Disease (PROCARDIS) study cohort showed that both *LPA* gene variants (rs3798220 and rs10455872) are associated with higher levels of Lp(a) (mg/dL) with odds ratios for coronary disease of 1.7 (rs10455872) and 1.92 (rs3798220). Nevertheless, after adjustment for the Lp(a) level, the association was abolished [14]. Although our study showed a strong association for only the *LPA* rs3798220 genotype, after an adjustment for Lp(a) levels the association was also eliminated. This could be explained by the smaller sample size in our study; however, this might also be explained since both groups (cases and controls) had elevated levels of Lp(a). Even though this was not the focus for this study, however, one limitation is that we did not include a cohort of healthy people without elevated Lp(a) to investigate the proportions of *LPA* genotypes.

The study of Pechlivanis et al. on a German population provided evidence for the association of *LPA* rs10455872 with higher Lp(a) levels and coronary artery calcification that leads to coronary disease, but for *LPA* rs3798220 it showed only an association with higher Lp(a) levels [27]. A study on a Mexican population also showed a strong association of the *LPA* rs10455872-*G* allele with higher levels of Lp(a) (up to 3.86 times) and the presence of aortic valve calcification (2.54 times), independently of other traditional risk factors [15]. It is interesting that our study did not show significant association for the *LPA* rs10455872 which is probably due to the smaller sample and the ethnicity of the subjects. On the other hand, a study of patients with possible familiar hypercholesterolemia (FH) showed that the *LPA* rs3798220-*C* allele is a significant predictor of coronary artery disease (CAD) independent of other traditional risk factors (T2DM, smoking status, ethnicity, etc.), while no association between the *LPA* rs10455872-*G* allele and CAD was found [28].

Although studies indicate an association of elevated Lp(a) levels with the development of aortic stenosis, in our study the association is negligible due to the small number of cases. Only 5% of patients from the case group had aortic stenosis recorded by a heart ultrasound. Given that patients who had recovered from a CVI performed a heart ultrasound as part of a regular check-up, the results are credible. On the other hand, no cases of aortic stenosis in the control group could be explained by irregular medical check-ups and a median age of 47 years. Another significant difference between our and other study results is the association of *LPA* variants with PAD. Most of studies found a positive association of the *LPA* gene variant with PAD [29,30]. Although studies suggest a higher likelihood of developing PAD with the rs3798220 variant, the overall risk may be influenced by other genetic and environmental factors [31]. On the other hand, our study identified the rs3798220-*C* allele with a decreased risk of PAD even after adjustment with Lp(a) levels. Given that only 23 subjects had PAD, the stated results should be interpreted with caution. Therefore, further research on a younger population with PAD is needed to establish a clear association with the rs3798220 variant.

According to the leading studies and guidelines, Lp(a) levels > 125 nmol/L, found in approximately 20% of the world’s population, significantly increase cardiovascular risk [32]. Considering the cost and inaccessibility of genetic testing, the main question that remains is, which patients should be tested for rs3798220-*C* allele? We provided optimal cut-off points for Lp(a) levels for cardiovascular outcome and based on family history of CVI. Our preliminary results suggest that patients with a positive family history of early CVI with Lp(a) levels > 151 nmol/L should be tested and evaluated for signs of ASCVD because of a higher risk for developing CVI. Also, patients with Lp(a) levels > 249 nmol/L (>99th percentile) should be genetically tested for high-risk SNP as there is a high chance of carrying the rs3798220 variant. However, in order to provide more accurate cut-off values, studies with a larger number of samples are needed. Therefore, we encourage researchers to take a similar approach in their future studies. We would also like to emphasize the importance of an early detection of homozygous patients for the minor allele (rs3798220-*CC*) that is rare [33], but accompanied by serious CVI, as in our young patient.

Possible limitations in our study include smaller sample size, selection bias (as in our cases are cardiology patients) and gene–environment interactions.

## 5. Conclusions

Our study provides evidence for the association of the *LPA* rs3798220-*C* allele (*TC* and *CC* genotypes) with higher Lp(a) levels and an increased risk for myocardial infarction at an early age of onset. However, the obtained association should further be evaluated at a much larger scale, and healthy individuals with normal Lp(a) levels should be included in order to study the LPA genotype proportions in more depth. The *LPA* rs10455872-*G* allele was not identified as a significant risk factor in our study on homogenous Croatian population subjects. According to the results from our study, we can recommend *LPA* genetic testing of patients with Lp(a) levels above 249 nmol/L, who could be carriers of the *LPA* rs3798220-*C* allele, in order to prevent or delay CVI and associated mortality.

## Figures and Tables

**Table 1:** Descriptive statistics of subjects with and without history of an early cardiovascular incident.

	Without CVI (N = 123)	With CVI (N = 128)	*p*
Gender, N (%)			
Women	65 (53)	41 (32)	0.001
Men	58 (47)	87 (67)	
Age, median (IQR)	47 (18)	57 (15)	<0.001
Lp(a), median (IQR)	189 (120.5)	228.5 (142)	<0.001
MI, N (%)	0	101 (79)	<0.001
ICS, N (%)	0	27 (21)	<0.001
PAD, N (%)	0	23 (18)	<0.001
AS, N (%)	0	7 (5)	0.025
T2DM, N (%)	10 (8)	26 (20)	0.010
Family history of early CVI, N (%)	97 (79)	101 (79)	1.000
*LPA* rs3798220 genotype, N (%)			
*TT*	100 (81.3)	91 (71.1)	0.123
*TC*	23 (18.7)	36 (28.1)	
*CC*	0	1 (0.8)	
*LPA* rs10455872 genotype, N (%)			
*AA*	81 (65.9)	90 (70.3)	0.534
*AG*	42 (34.2)	38 (29.7)	
*GG*	0	0	
*LPA* rs3798220 + *LPA* rs10455872 genotypes, N (%)			
rs3798220*-TT +* rs10455872*-AA*	61 (49.6)	58 (45.7)	0.336
rs3798220*-TT +* rs10455872*-AG*	39 (31.7)	33 (26.0)	
rs3798220*-TC +* rs10455872*-AA*	20 (16.3)	31 (24.4)	
rs3798220*-TC +* rs10455872*-AG*	3 (2.4)	5 (3.9)	

Abbreviations: AS—aortic stenosis; CVI—cardiovascular incident; ICS—ischemic cerebral stroke; Lp(a)—lipoprotein(a); MI—myocardial infarction; PAD—peripheral arterial disease; T2DM—type 2 diabetes mellitus.

**Table 2:** Results of different logistic regression models for cardiovascular incident, myocardial infarction and peripheral arterial disease.

Model	Independent Variable	OR (95% CI)	*p*
Outcome: Cardiovascular incident			
Model 1: univariate logistic regression	*LPA* rs3798220-*C*	1.79 (1.00–3.21)	0.049
Model 1: univariate logistic regression, age-adjusted	*LPA* rs3798220-*C*	1.92 (1.04–3.55)	0.037
Model 1: univariate logistic regression	*LPA* rs10455872-*G*	0.81 (0.48–1.39)	0.449
Model 1: univariate logistic regression, age-adjusted	*LPA* rs10455872-*G*	0.78 (0.44–1.36)	0.376
Model 2: multivariate logistic regression	*LPA* rs3798220-*C*	1.28 (0.67–2.44)	0.461
	*LPA* rs10455872-*G*	0.86 (0.49–1.50)	0.585
	Lp(a), by every 10 nmol/L **	1.03 (1.01–1.06)	0.006
Model 2: multivariate logistic regression, age-adjusted	*LPA* rs3798220-*C*	1.35 (0.68–2.67)	0.391
	*LPA* rs10455872-*G*	0.82 (0.46–1.49)	0.521
	Lp(a), by every 10 nmol/L **	1.03 (1.01–1.06)	0.009
Model 3: stepwise multivariate logistic regression *	Male gender	3.87 (2.11–7.10)	<0.001
	Age	1.08 (1.05–1.11)	<0.001
	Lp(a), by every 10 nmol/L **	1.04 (1.02–1.07)	0.001
Outcome: Myocardial infarction			
Model 1: univariate logistic regression	*LPA* rs3798220-*C*	2.05 (1.16–3.64)	0.014
Model 1: univariate logistic regression, age-adjusted	*LPA* rs3798220-*C*	2.20 (1.2–4.01)	0.010
Model 1: univariate logistic regression	*LPA* rs10455872-*G*	0.72 (0.42–1.25)	0.248
Model 1: univariate logistic regression, age-adjusted	*LPA* rs10455872-*G*	0.69 (0.39–1.22)	0.199
Model 2: multivariate logistic regression	*LPA* rs3798220-*C*	1.62 (0.86–3.05)	0.132
	*LPA* rs10455872-*G*	0.80 (0.45–1.42)	0.451
	Lp(a), by every 10 nmol/L **	1.02 (1.00–1.04)	0.082
Model 2: multivariate logistic regression, age-adjusted	*LPA* rs3798220-*C*	1.73 (0.9–3.34)	0.102
	*LPA* rs10455872-*G*	0.78 (0.43–1.41)	0.405
	Lp(a), by every 10 nmol/L **	1.02 (1.00–1.04)	0.103
Model 3: stepwise multivariate logistic regression *	Male gender	5.10 (2.72–9.53)	<0.001
	Age	1.08 (1.05–1.11)	<0.001
	Lp(a), by every 10 nmol/L **	1.03 (1.00–1.05)	0.031
	*LPA* rs3798220-*C*	1.69 (0.86–3.36)	0.131
Outcome: Peripheral arterial disease			
Model 1: univariate logistic regression	*LPA* rs3798220-*C*	0.28 (0.06–1.23)	0.091
Model 1: univariate logistic regression, age-adjusted	*LPA* rs3798220-*C*	0.27 (0.06–1.19)	0.083
Model 1: univariate logistic regression	*LPA* rs10455872-*G*	0.73 (0.28–1.94)	0.533
Model 1: univariate logistic regression, age-adjusted	*LPA* rs10455872-*G*	0.72 (0.27–1.91)	0.505
Model 2: multivariate logistic regression	*LPA* rs3798220-*C*	0.17 (0.04–0.78)	0.023
	*LPA* rs10455872-*G*	0.55 (0.20–1.50)	0.244
	Lp(a), by every 10 nmol/L **	1.04 (1.01–1.07)	0.019
Model 2: multivariate logistic regression, age-adjusted	*LPA* rs3798220-*C*	0.16 (0.03–0.75)	0.021
	*LPA* rs10455872-*G*	0.52 (0.19–1.44)	0.209
	Lp(a), by every 10 nmol/L **	1.04 (1.01–1.07)	0.022
Model 3: stepwise multivariate logistic regression *	Age	1.04 (0.99–1.09)	0.117
	Lp(a), by every 10 nmol/L **	1.04 (1.01–1.07)	0.020
	T2DM	2.43 (0.85–6.93)	0.098
	*LPA* rs3798220-*C*	0.19 (0.04–0.89)	0.035

* The stepwise multivariate logistic regression included the following independent variables: gender, age, Lp(a) levels, presence of aortic stenosis, presence of type 2 diabetes mellitus, family history of early cardiovascular incidents, *LPA* rs3798220-*C* and *LPA* rs10455872-*G*. ** To obtain interpretable 95% CI, we have transformed the Lp(a) concentration levels where the unit of change was set to 10 nmol/L (instead of 1 nmol/L) due to the large range of Lp(a) concentrations (range from 79.0 to 800.0 nmol/L). Abbreviations: Lp(a)—lipoprotein(a); T2DM—type 2 diabetes mellitus.

**Table 3:** Optimal cut-off points for Lp(a) levels with increased risk for cardiovascular incident, myocardial infarction or peripheral arterial disease.

Stratification	Lp(a) Optimal Cutpoint	Sensitivity	Specificity	AUC
Outcome: Cardiovascular incident				
/	152	0.844	0.366	0.628
*LPA* rs3798220—*TT*	152	0.813	0.390	0.605
*LPA* rs3798220—*TC*	149	0.944	0.261	0.612
No family history of CVIs	217	0.556	0.769	0.620
Family history of CVIs	151	0.881	0.361	0.634
Outcome: Myocardial infarction				
/	243	0.475	0.713	0.599
*LPA* rs3798220—*TT*	152	0.797	0.344	0.544
*LPA* rs3798220—*TC*	249	0.774	0.536	0.651
No family history of CVIs	217	0.522	0.700	0.564
Family history of CVIs	151	0.897	0.325	0.606
Outcome: Peripheral arterial disease				
/	165	0.957	0.355	0.612
*LPA* rs3798220—*TT*	165	0.952	0.424	0.668
*LPA* rs3798220—*TC*	537	0.500	0.930	0.675
No family history of CVIs	182	1.000	0.531	0.712
Family history of CVIs	157	1.000	0.285	0.590

Abbreviations: CVI—cardiovascular incident, AUC—area under the curve.

## Data Availability

Data are available upon request.

## Supplementary Materials

The following supporting information can be downloaded at https://www.mdpi.com/article/10.3390/diagnostics15040404/s1, Figure S1: Power analysis of study.

## Abbreviations

The following abbreviations are used in this manuscript:

AS	Aortic stenosis
ASCVD	Atherosclerotic cardiovascular disease
CAD	Coronary artery disease
CRP	C-reactive protein
CVD	Cardiovascular disease
CVI	Cardiovascular incident
ESR	Erythrocyte sedimentation rate
FH	Familial hypercholesterolemia
ICS	Ischemic cerebral stroke
IQR	Interquartile range
KIV-2	The kringle IV type-2
LDL	Low-density lipoprotein
LDLR	Low-density lipoprotein receptor
Lp(a)	Lipoprotein (a)
MI	Myocardial infarction
PAD	Peripheral arterial disease
PCSK-9	Proprotein convertase subtilisin/kexin type 9
RNA	Ribonucleic acid
SNP	Single nucleotide polymorphism
T2DM	Type 2 diabetes mellitus
VLDLR	Very-low-density-lipoprotein receptor
