# Supplementary material for: Association of rs3798220 Polymorphism with Cardiovascular Incidents in Individuals with Elevated Lp(a)
Source: Diagnostics (Basel). 2025 Feb 7;15(4):404. doi: 10.3390/diagnostics15040404 (PMC11854902; doi:10.3390/diagnostics15040404)
Supplement: Supplementary file 1 [file diagnostics-15-00404-s001.zip › diagnostics-3430746-supplementary.pdf]

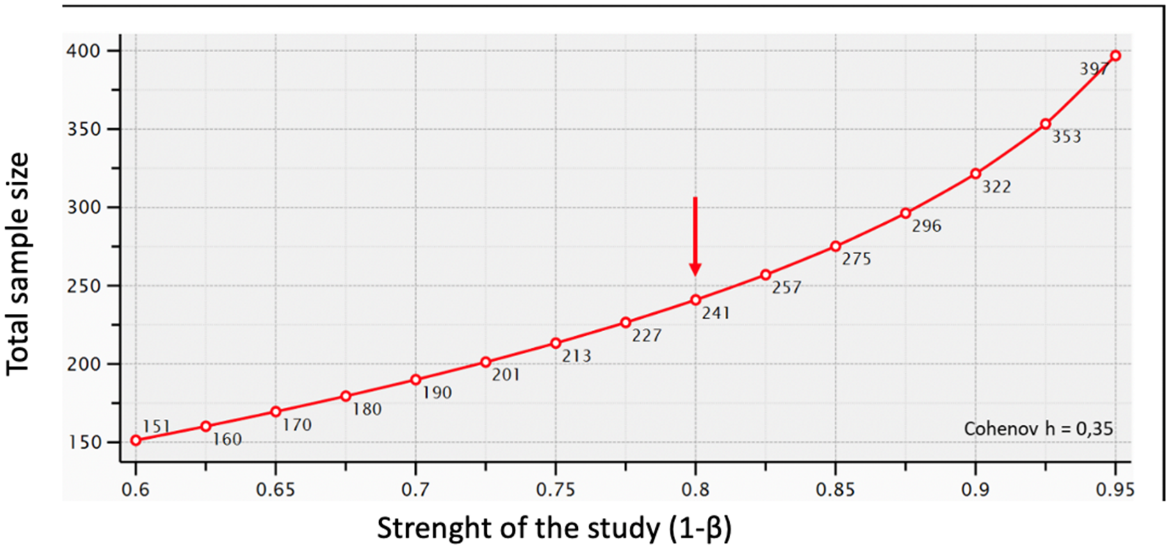

Figure S1: Power analysis of study

The number of respondents was determined on the basis of an a priori analysis in which, with the power of the study of 0.8, the level of statistical significance $\alpha=0.05$, and the value of the effect size between low and medium levels, it was determined that 241 respondents should be included in the study, which is acceptable for obtaining relevant data
